# In Planta Preliminary Screening of ER Glycoprotein Folding Quality Control (ERQC) Modulators

**DOI:** 10.3390/ijms19072135

**Published:** 2018-07-23

**Authors:** Lucia Marti, Andrea Lia, Ida-Barbara Reca, Pietro Roversi, Angelo Santino, Nicole Zitzmann

**Affiliations:** 1 Institute of Sciences of Food Production, C.N.R. Unit of Lecce, via Monteroni, I-73100 Lecce, Italy; luciamarti.lm@gmail.com (L.M.); andrea.lia.lecce@gmail.com (A.L.); recabarbara@yahoo.it (I.-B.R.); 2 Oxford Glycobiology Institute, Department of Biochemistry, University of Oxford, South Parks Road, Oxford OX1 3QU, UK; 3 Leicester Institute of Structural and Chemical Biology, Department of Molecular and Cell Biology, University of Leicester, Henry Wellcome Building, Lancaster Road, Leicester LE1 7RH, UK

**Keywords:** endoplasmic reticulum, glycoprotein folding quality control, *Arabidopsis thaliana*, drug screening, iminosugar, *N*B-DNJ, plant immune response

## Abstract

Small molecule modulators of the Endoplasmic Reticulum glycoprotein folding quality control (ERQC) machinery have broad-spectrum antiviral activity against a number of enveloped viruses and have the potential to rescue secretion of misfolded but active glycoproteins in rare diseases. In vivo assays of candidate inhibitors in mammals are expensive and cannot be afforded at the preliminary stages of drug development programs. The strong conservation of the ERQC machinery across eukaryotes makes transgenic plants an attractive system for low-cost, easy and fast proof-of-concept screening of candidate ERQC inhibitors. The *Arabidopsis thaliana* immune response is mediated by glycoproteins, the folding of which is controlled by ERQC. We have used the plant response to bacterial peptides as a means of assaying an ERQC inhibitor in vivo. We show that the treatment of the plant with the iminosugar *N*B-DNJ, which is a known ER α-glucosidase inhibitor in mammals, influences the immune response of the plant to the bacterial peptide elf18 but not to the flagellin-derived flg22 peptide. In the *N*B-DNJ-treated plant, the responses to elf18 and flg22 treatments closely follow the ones observed for the ER α-glucosidase II impaired plant, *At psl5-1*. We propose *Arabidopsis thaliana* as a promising platform for the development of low-cost proof-of-concept in vivo ERQC modulation.

## 1. Introduction

Eukaryotic glycoproteins in the cellular secretory pathway require the endoplasmic reticulum (ER) protein-folding quality control (ERQC) machinery in order to fold correctly [1]. Small molecule modulators that target the ERQC α-glucosidases hamper glycoprotein entry into and/or exit from the ER glycoprotein folding cycle [2,3]. Such inhibitors have broad-spectrum antiviral activity against a number of enveloped viruses and have the potential to rescue the secretion of misfolded but active glycoproteins in rare diseases [4,5]. The strong conservation of the ERQC machinery across eukaryotes makes plants an attractive system for low-cost, easy and fast screening of candidate ERQC modulators, much in the same way as plants sensitive to the action of human chemotherapeutics have been used for preliminary screening of anti-cancer drugs efficacy [6]. The best-known class of such ERQC modulators are iminosugars, glucose analogues that have the potential to act as broad-spectrum antivirals. Iminosugars target the host’s ERQC and negatively affect the folding of a number of enveloped viral glycoproteins [7,8,9,10,11]. The cross reactivity of iminosugars with intestinal glucosidases and enzymes of the glycosphingolipid pathway [12] highlights the need to expand the search for ERQC modulators to different scaffolds. In vitro screens have been proposed to offer several advantages over phenotype-based in vivo methods as a starting point for drug repositioning [13] but modulators of the activity of ER α-glucosidases need to cross both the plasma- and ER-membranes. This challenge makes cheap and easy in vivo screening a more appealing strategy towards the development of compounds that possess both activity and the ability to reach their cellular targets in the ER lumen.

In order to develop an in vivo assay for ERQC inhibition by candidate molecules, it is necessary to relate their activity to an observable phenotype that depends on ERQC. *Arabidopsis thaliana* plants (Columbia-0 ecotype, Col-0) recognize characteristic bacterial structures, such as the N-terminus of bacterial elongation factor Tu (EF-Tu) and flagellin [14,15]. The N-acetylated peptide elf18 comprises the first 18 amino acids of EF-Tu, while the flg22 peptide spans 22 conserved amino-acids of bacterial flagellin. The response to elf18 is mediated by the EF-Tu receptor (EFR) [16] and the response to flg22 is mediated by the Flagellin *S*ensitive *2* (FLS2) receptor [17]. Both EFR and FLS2 are secreted glycoproteins that reach the plant cell membrane after attaining their native folding in the ER. Accumulation and signalling of EFR (but not of FLS2) are impaired in the plant by mutations in the genes coding for calreticulin3 and UGGT [18], both of which ERQC proteins. Similarly, plants carrying mutations in the genes coding for the two subunits of ER α-glucosidase II (α-GluII, the central ERQC enzyme) are insensitive to elf18 but responsive to flg22 [19]. Thus, the plant response to elf18 can be used to monitor the folding of the EFR receptor, which in turn depends on ER α-glucosidase II activity, with the flg22 treatment providing a negative control.

Here, we set out to exploit the *Arabidopsis thaliana* immune response to bacterial peptides [19] as a means of assaying iminosugar activity in the ER in vivo. We show that treatment with the known ER α-glucosidase iminosugar inhibitor *N*B-DNJ influences the immune response of the plant to the bacterial EF-Tu peptide elf18 but not to the bacterial flagellin-derived flg22 peptide. Importantly, in *N*B-DNJ treated plants, the phenotype closely follows the one observed for the ER α-glucosidase II impaired plant, *At psl5-1* [19]. This is the first observation that an iminosugar, which has reached clinical trial phase I as a mammalian ER α-glucosidase II inhibitor [20], also likely inhibits the plant enzyme. Strictly speaking, we cannot rule out the possibility that the molecule interferes with either other secretory pathway components causing mis-folding and/or mis-localization of elf18-receptor, or with any of the components of the elf18 response.

## 2. Results

### 2.1. The Iminosugar NB-DNJ Is Toxic to Arabidopsis thaliana Plants at Concentrations above 200 µM

**Figure 1:**
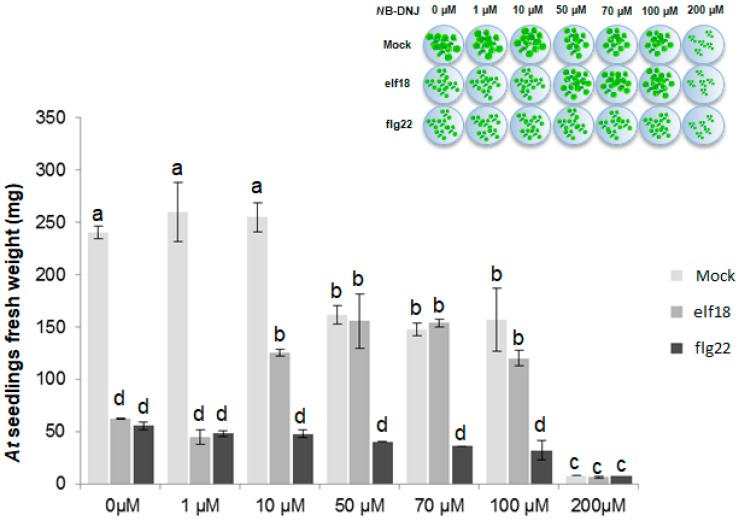
*N*B-DNJ toxicity assay. *Arabidopsis thaliana* (*At*) seedlings, Col-0 ecotype, were grown in Murashige and Skoog (MS) half-strength liquid medium containing different concentrations of *N*B-DNJ in combination with 100 nM of elf18 or flg22 (inset). After 15 days of treatment, the fresh weight of seedlings was measured. Increasing concentrations of *N*B-DNJ resulted in increasing reduction in plant growth, with severe weight loss at 200 µM of *N*B-DNJ. Values represent the mean of at least five independent experiment (± s.e.; *n* = 5) with similar results in each experiment. Each letter (a, b, c and d) indicates a group of samples whose values are statistically equivalent to the ones in the same group, at the (*p* < 0.01) level determined by ANOVA with Scheffè’s *post hoc* test.

The iminosugar *N*B-DNJ [20,21] is well-tolerated in humans, with concentrations as high as 1 mg/mL (around 5 mM) displaying no cytotoxicity [22]. To test the toxicity of *N*B-DNJ in *Arabidopsis thaliana*, 15-day-old plants were treated with increasing concentrations of inhibitor, on the basis of a previously developed growth inhibition assay [17]. In the mock treatment experiments in Figure 1, the impact of *N*B-DNJ treatment on plant growth was measured. Arrest of wild-type seedling growth at *N*B-DNJ concentrations higher than 200 µM indicated toxicity. At lower *N*B-DNJ concentrations (1 and 10 µM), there was no impact on seedling growth. Supplementation of *N*B-DNJ at the intermediate concentrations of 50 µM, 70 µM and 100 µM resulted in reduced plant growth (about 30–40% of wild-type), with clear differences between elf18- and flg22-treated plants at the same concentrations. The concentration of 70 µM *N*B-DNJ was chosen to monitor α-Glu II activity.

### 2.2. NB-DNJ Doses Higher than 50 µM Render Arabidopsis thaliana Plants Insensitive to the elf18 Peptide

ER α-glucosidase II admits its client glycoproteins to the calnexin cycle and the mutations in the gene encoding it (*At PSL5* in the plant) can cause client glycoprotein misfolding and loss of function. *At* ER α-Glu II impairment can be probed in the plant by studying the response to bacterial peptides elf18 and flg22. The *At psl5-1* mutant carries a DNA mutation causing the S517F point substitution in the *At* ER α-glucosidase II α-subunit. The mutant plant is insensitive to elf18, suggesting that ER α-Glu II activity/inhibition can be monitored by measuring the responses induced by elf18 treatment [14]. In those experiments, flg22 treatment was used as a control because the response of the plant to this peptide is independent of ER α-Glu II activity [19]. The published data suggest that either EFR or another protein in the elf18 signalling pathway or involved in EFR localisation (but not FLS2 nor any proteins involved in flg22 signalling pathway/localisation) fold under ER α-glucosidase II control.

**Figure 2:**
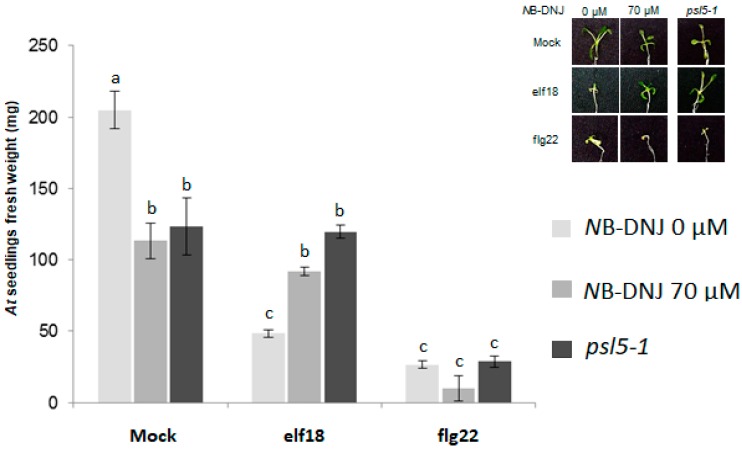
In planta ER α–glucosidase II assay. *Arabidopsis thaliana* (*At*) Col-0 seedlings (wild-type, WT) were grown in presence or absence of *N*B-DNJ (70 µM) and their growth was compared to that of *psl5-1* seedlings used as controls. *N*B-DNJ treatment was combined with that of elf18 or flg22. In presence of *N*B-DNJ, the growth trend of *psl5-1* control seedlings was indistinguishable from WT ones, irrespective of treatment with elicitors. Inset shows representative pictures of 15-day-old seedlings (five seedlings per well) germinated and grown with or without *N*B-DNJ and elicitors. Values represent the mean of at least five independent experiments (± s.e.; *n* = 5) with similar results in each experiment. Each letter indicates a group of samples whose values are statistically equivalent to the ones in the same group, at the (*p* < 0.01) level determined by ANOVA with Scheffè’s *post hoc* test. Note: the differences in the weights of seedlings from those in the experiment in Figure 1, which may be due to slight variations in seed germination times (see methods), did not affect the sensitivity of plants to elicitors after *N*B-DNJ treatment.

To check if *N*B-DNJ inhibits *At* ER α-glucosidase II in vivo, we supplemented *A*. *thaliana* plants with *N*B-DNJ at concentrations of 70 µM and treated the same plants with elf18 and flg22. Iminosugar treatment resulted in significant resistance to elf18 but not to flg22 (Figure 2). These *N*B-DNJ-induced elf18- and flg22-response phenotypes are similar to the ones of the *At psl5-1* mutant plant with the same elicitors [19]. Taken together, our data suggest that *N*B-DNJ inhibits *At* ER α-glucosidase II in vivo at 70 µM.

**Figure 3:**
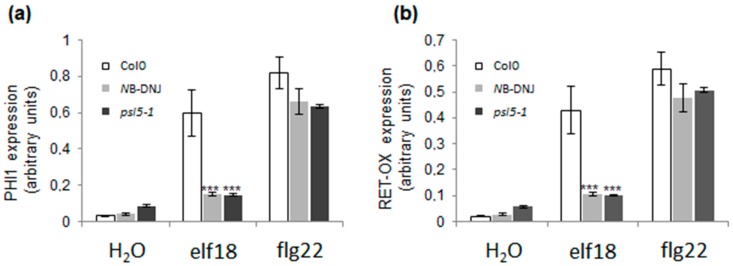
Transcription of defense genes. *Arabidopsis thaliana* WT (wild-type, empty bars), WT grown in the presence of *N*B-DNJ (70 µM, light grey bars) and *psl5-1* (dark grey bars) 10-day-old seedlings (20 seedlings in each experiment) were treated with water or elf18 and flg22 elicitors. Transcription of *PHI1* and *RET-OX* genes were determined by qRT-PCR 30 min after treatment. Transcription levels are shown as the mean of at least three independent experiments (± s.e.; *n* = 3) normalized to *UBQ5* (ubiquitin 5) used as a reference. In both (**a**) and (**b**), asterisks indicate statistically significant differences between elf18-treated seedlings (WT + *N*B-DNJ and *psl5-1*) and corresponding treatment of the wild type according to Student’s *t*-test (3 asterisks = *p* < 0.001).

To confirm that the defect in elf18 response is accompanied by reduced defense signaling, we investigated the transcription levels of two genes that are typically induced by elf18 and flg22. *Phosphate-induced 1* (*PHI1*) and *Reticulin-oxidase homologue* (*RET-OX*) are indicated as early elicitor-induced genes because their transcription is maximal within 1 h after elicitor treatment [23]. Ten-day-old Col-0 seedlings, grown in absence or presence of 70 µM of *N*B-DNJ, and *psl5-1* seedlings were treated with elf18 (or flg22, as a control) for 30 min. Low transcription of *PHI1* and *RET-OX* in *N*B-DNJ treated seedlings were comparable with those recorded in the *psl5-1* mutant in response to elf18 (Figure 3). Conversely, flg22 treated seedlings showed a normal defense response characterized by higher transcription levels of *PHI1* and *RET-OX* (Figure 3).

### 2.3. Treatment with 70 µM NB-DNJ Is Lethal to the At psl5-1 Mutant

ERQC plays a vital role in the development of multicellular organisms [24]. For example, a crucial developmental role of the ERQC checkpoint UGGT1 was demonstrated in mice embryos [25]. Furthermore, UGGT mutant plants show altered growth rates during vegetative development compared to wild type [26] although the *UGGT* gene can be knocked out in *S. pombe* without impairing cell growth [27]. Both known *At* ER α-glucosidase II missense mutations (the *At psl5-1* S517F mutant and the *At rsw3* S599F mutant) grow in a similar way to wild type [19], which raises questions as to the residual activity of the mutated proteins.

**Figure 4:**
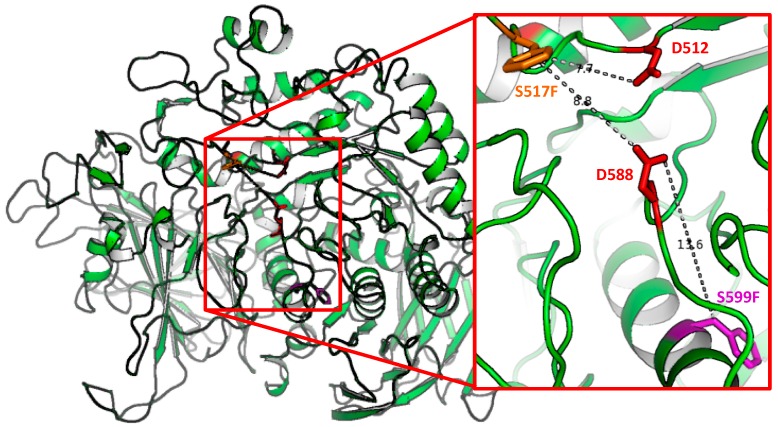
Structural mapping of the *psl5-1* and *rsw3* missense mutations in *At* ER α-glucosidase II. Homology model of the *At* ER α-glucosidase II α subunit in green cartoon representation. The inset shows the *At psl5-1* mutant S517F point substitution (orange sticks), and the *At rsw3* mutant S599F substitution (magenta sticks). The catalytic Asp residue D512 and the acid–base catalyst residue D588 are depicted in red sticks. The distances between the mutated residues and the catalytic residues are indicated by dotted lines, with the distances in Å. Both the S517F and the S599F mutations are likely to destabilise the catalytic domain of the enzyme.

Homology modelling of the *At* ER α-glucosidase II protein, based on the recently determined crystal structure of the mouse enzyme [20], was used to gain insight on the reasons why the S517F and S599F point mutations affect *At* ER α–glucosidase II activity. In the *At psl5-1* ER α-glucosidase II homology model (Figure 4), Ser517 is located about 8 Å from the nucleophilic catalytic residue Asp512 and about 9 Å from the catalytic acid/base Asp588 (Figure 4, inset). The *At rsw3* S599F mutation is further away from the active site (Figure 4, inset). Neither mutant directly affects the catalytic residues of the enzyme. The possibility that either mutant carries residual ER α–glucosidase II activity cannot be ruled out.

**Figure 5:**
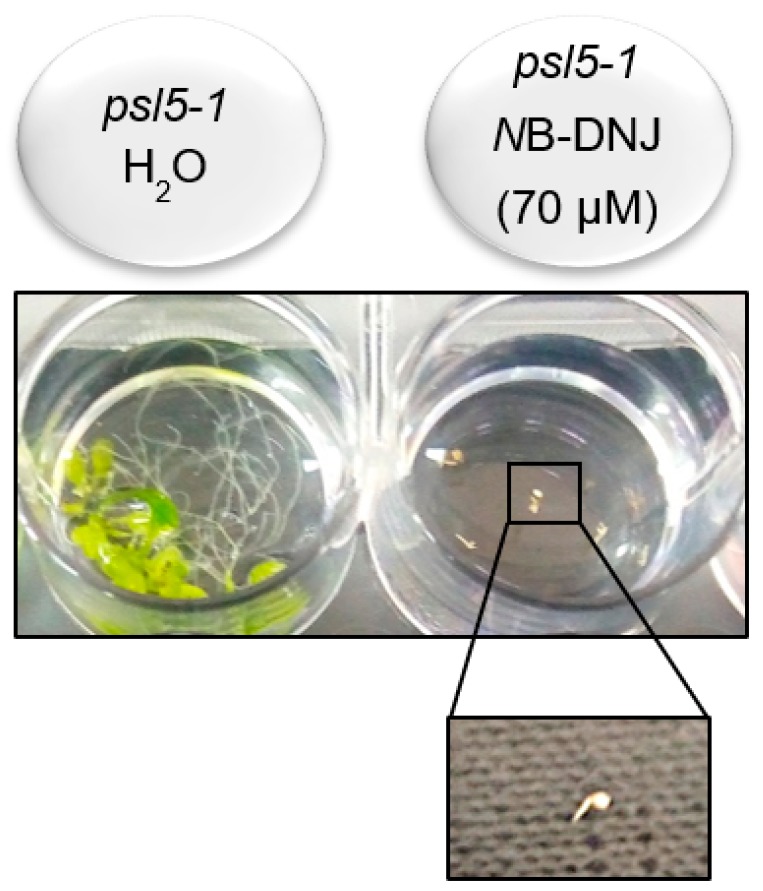
*At psl5-1* and *N*B-DNJ. *Arabidopsis thaliana psl5-1* seedlings show impaired growth in presence of 70 µM *N*B-DNJ.

To check if we could inhibit the putative residual *At* ER α-glucosidase II activity in *At psl5-1*, we grew *At psl5-1* mutants in the presence of 70 µM *N*B-DNJ. Under these experimental conditions, the growth of germinated embryos is completely impaired and treatment with 70 µM *N*B-DNJ is lethal to the *At psl5-1* mutant (Figure 5). As a result, we could not carry out experiments with the elf18 and flg22 peptides in the *At psl5-1* plant.

## 3. Discussion

Iminosugars, currently the best known broad-spectrum antivirals, show cross-reactivity with intestinal glucosidases and enzymes of the glycosphingolipid pathway [12], creating a need to expand the search for new ERQC modulators. The development of low-cost platforms for the screening of molecules with potential pharmaceutical effects before testing them on animal models is only just emerging [28]. In this context, plants have been already proposed for preliminary screening of anti-cancer compounds [3]. The ERQC machinery is strongly conserved across eukaryotes, including plants, and many KO/mutant plants exist which can be used as negative controls; combined with the plant’s well characterised response to bacterial elicitors of the EFR/FLS2 receptors, it is clear that plants potentially afford a low-cost, easy and fast screening platform of ERQC modulators in vivo. In this work, we show that the ER α-glucosidase II inhibitor *N*B-DNJ influences the immune response of the plant to the bacterial peptide elf18, with a phenotype similar to the ER α-glucosidase II impaired plant, *At psl5-1*. The same iminosugar does not impair the plant response flagellin-derived flg22 peptide, again similarly to what observed in *At psl5-1*. This is the first observation that an iminosugar, which has reached clinical trial phase I as a mammalian ER α-glucosidase II inhibitor [20], also likely inhibits the plant enzyme in vivo. Alternatively, it is still conceivable that the molecule may act by interfering with either the trafficking or folding of the elf18-receptor and/or other proteins that are located downstream and involved in the elf18 signalling pathway.

Given the strong conservation of ERQC sequence and function across eukaryotes, the study strongly supports further efforts towards the development of the plant as a low-cost platform for early stage assays of ERQC modulators.

## 4. Materials and Methods 

### 4.1. Plant Material, Growth Conditions and Treatments

For the growth inhibition assay, the seeds were surfaced, sterilized and sown in multi-well plates containing one-half strength Murashige and Skoog medium [29] (five seeds per 2 mL of liquid medium in the wells of 12-well-plates) supplemented with 0.5% (*w*/*v*) of sucrose. For *N*B-DNJ treatment, the seeds were germinated and grown in one-half-strength Murashige and Skoog medium containing the iminosugar *N*B-DNJ. After germination (3 day-post sowing), the medium was supplied with elicitors. The effect of treatment with the different peptides on seedling growth was analysed 15 days post sowing by photography and/or weighing (fresh weight). Variations in the weight of seedlings due to seed germination times that were not perfectly synchronized under slightly different environmental conditions (i.e., growth chamber temperature and light) did not affect the sensitivity of plants to elicitors after *N*B-DNJ treatment.

Elicitor treatment of seedlings was performed using elf18 (ac-SKEKFERTKPHVNVGTIG) and flg22 (QRLSTGSRINSAKDDAAGLQIA) at a concentration of 100 nM. Seedlings were grown at 22 °C and 70% relative humidity under a 16-h-light/8-h-dark cycle (approximately 120 µmol m^–2^ s^–1^). For gene transcription analysis, the seeds were surfaced, sterilized and sown in multi-well plates (approximately 10 seeds per well) containing one-half strength Murashige and Skoog medium (2 mL per well). After 9 days, the incubation medium was replaced with fresh medium, before the treatments with elicitors were performed after 24 h.

### 4.2. Homology Modelling 

The protein sequences of the *At* ER α-glucosidase II α and β subunits were taken from the Uniprot database entries PSL5_ARATH and PSL4_ARATH, respectively. The sequences were aligned with the sequences of the mouse ER α-glucosidase II α and β subunits using Clustal omega [30]. The alignment was used in the program Modeller [31] together with PDB entry 5F0E, in order to produce a homology model of *At* ER α-glucosidase II α subunit residues 22–921 and β subunit residues 52–134. The *At* ER α-glucosidase II α *psl5-1* S517F and *rsw3* S599F point substitutions were modelled in PyMOL [32].

### 4.3. Gene Transcription Analysis

For gene transcription analysis, the seedlings were frozen in liquid nitrogen and homogenized with a MM301 Ball Mill (Retsch, Haan, Germany). Total RNA was extracted from at least two independent replicates, each having 20 seedlings, with NucleoZol reagent (Machery-Nagel, Duren, Germany) according to the manufacturer’s protocol. Total RNA (2 µg) was treated with RQ1 DNase (Promega, Madison, WI, USA), and the first-strand complementary DNA was synthesized using ImProm-II reverse transcriptase (Promega) according to the manufacturer’s instructions. qRT-PCR analysis was performed by using a CFX96 Real-Time System (Bio-Rad, Hercules, CA, USA). Complementary DNA (corresponding to 50 ng of total RNA) was amplified in a 20-µL reaction mix containing 1X GoTaq Real-Time PCR System (Promega) and 0.4 µM of each primer. Three technical replicates were performed for each sample, before data analysis was conducted using LinRegPCR software (Ruijter, Amsterdam, Netherlands). The transcription levels of each gene relative to *UBIQUITIN5* were determined using a modification of the Pfaffl method [33] as described in reference [34] and expressed in arbitrary units. Primer sequences are shown in Table 1.

### 4.4. Statistical Analysis

The average weight of five plants per treatment was compared and statistically analyzed using ANOVA with Scheffè’s *post hoc* test. The statistical analysis on gene transcription data was performed using the Student’s *t*-test.

## Figures and Tables

**Table 1:** Primers used in this work.

GENE	AG CODE	FORWARD PRIMER (5′-3′)	REVERSE PRIMER (5′-3′)
*UBQ5*	AT3G62250	GGAATCGACGCTTCATCTCG	ATGAAAGTCCCAGCTCCACA
*PHI1*	AT1G35140	TTGGTTTAGACGGGATGGTG	ACTCCAGTACAAGCCGATCC
*RET-OX*	AT1G26380	AGGTTCTCGAACCCTAACAACA	GCACAGACGACACGTAAGAAAG
